# Fictitious Catalogues of Medical Students.—Hostility to Medical Schools

**Published:** 1846-03-01

**Authors:** 


*Fictitious Catalogues of Medical Students—Hostility to Medical Schools.*—A correspondent of the Boston Med. and Surg. Journal has promised to furnish instances in which medical institutions have issued fictitious catalogues, for the purpose of magnifying their importance and attracting students. We have frequently heard it intimated that considerable latitude in this respect is sometimes indulged,—for example recognising as pupils, those who casually drop in at lectures, and those who take tickets for a single branch only, without any reference to becoming practitioners ; but we must confess astonishment that even a single institution has been so recreant to principles of common honesty as deliberately to coin names, representing men of straw, to enumerate in its catalogue. We sincerely trust that the correspondent of the Boston Journal, if he have facts sustaining his assertions, will publish them, directing attention specifically to the school or schools which are guilty of such knavery. We shall be happy to republish authentic statements exposing any disgraceful practices of medical schools, and to contribute in so far as we are able, to hold them up to the merited contempt of the intelligent and high minded members of the profession.

In making these remarks we would take occasion to add that we have no sympathy with those who seem to have engaged in a crusade against medical schools. It has become fashionable of late with some to consider them the light of scape-goats for all the evils which oppress the profession. We may be said to have in our state an Anti-medical school party, and we have been informed by a delegate that considerable feeling was manifested on the subject at the last meeting of the State Society. But we would ask the advocates of a destructive policy, have medical schools been of no service ? Have they not effected good ; and are they not still doing good ? Granting that they are not what could be desired—where lies the fault ? Does it lie with those, who, with but little public aid, and often, at considerable pecuniary sacrifice, to say nothing of time and labor, have established schools, and made them what they are ? We do not doubt that the officers and teachers in the majority, at least, of our medical institutions are as disinterestedly solicitous to elevate the standard of professional requirement as are any portion of the profession. We believe that the medical teachers of our country, as a body, are high-minded men, imbued with an earnest disposition to labor for the best interests of the profession. Assuredly the office of teacher does not afford a very wide field for selfish or mercenary motives. Poorly as ordinary professional services are remunerated, those of a faithful, deserving teacher, generally receive a less proportional compensation. Admit that candidates for the office of teacher allow ambitious motives to mingle with their aspirations of usefulness—is this to be quarreled with, and have they any reason to be ashamed of it ? Let those whose concern for the welfare of the profession has given rise to hostility to medical schools, reflect upon these and other things. Let them ask themselves what substitutes are at hand, provided their opposition should be effectual. That imperfections and abuses exist none will deny: let these persons unite to correct, reform and improve, and more good will result than from their efforts to injure or destroy.

Medical Journals should hold independent, impartial positions in these, as in all other matters. Let instances of malversation be communicated through them. We cheerfully offer our pages for the publication of authenticated facts from responsible individuals on this subject. Should the correspondent of our contemporary, the Boston Journal, sustain his allegations, we will not fail to apprise our readers of it, and republish all particulars.

